# New record of a parasitising species of *Hydrachna* (Acari, Hydrachnidia) on water beetles *Eretesgriseus* (Fabricius, 1781) (Coleoptera, Dytiscidae, Dytiscinae, Eretini)

**DOI:** 10.3897/zookeys.865.34532

**Published:** 2019-07-22

**Authors:** Elham Arjomandi, Andrzej Zawal, Hamidreza Hajiqanbar, Ewa Filip, Magdalena Szenejko

**Affiliations:** 1 Department of Entomology, Faculty of Agriculture, Tarbiat Modares University, 14115-336, Tehran, Iran Tarbiat Modares University Tehran Iran; 2 Department of Invertebrate Zoology and Limnology, University of Szczecin, Waska 13, 71-415 Szczecin, Poland University of Szczecin Szczecin Poland; 3 Department of Molecular Biology and Cytology University of Szczecin, Waska 13, 71-415 Szczecin, Poland Tarbiat Modares University Tehran Iran; 4 Department of Ecology and Environmental Protection, University of Szczecin, Waska 13, 71-415 Szczecin, Poland University of Szczecin Szczecin Poland

**Keywords:** Iran, larva, morphological features, taxonomic status, water beetles, water mites

## Abstract

The larvae of water mites of the genus *Hydrachna* parasitise water bugs and water beetles. Larvae of the genus *Hydrachna* attach to the thorax and abdomen sternites and tergites under the elytra. Up to now six species of *Hydrachna* were recorded from Iran, but there are no records on larvae parasitising on water beetles. There is some information about parasitising of *Hydrachna* on water beetles from the genus *Eretes*, which is very well adapted to dry climate. The aim of this paper is to describe the morphology of an unknown larva of the genus *Hydrachna*, found on *Eretesgriseus*.

## Introduction

Many organisms are dependent on a living host for some part of their life cycle or even the whole life. These symbiotic relationships categorised as mutualistic, commensal or parasitic, while specific specialisation occurs in commensalism and mutualistic symbionts, higher levels of co-evolution can be found in parasitic relationships. Among different groups of mites, larval stages of the cohort Parasitengonina parasitise a wide range of arthropods including terrestrial, freshwater or marine insects (Zawal 2003a; Baker et al. 2008; Normant et al. 2013; Mortazavi et al. 2018).

Beetles of the genus *Eretes* are specifically adapted to desert environments and a dry climate, where they can find small and isolated, warm, standing, water sources such as pools with clayey or sandy bottom and little vegetation (Hájek et al. 2014; Miller 2002). The water mites of subcohort Hydrachnidiae are well known as parasites of water beetles. Larval instars of the genus *Acherontacarus* Angellier attach to the mesosternal region of their host beetles (Aykut et al. 2018), larvae of the genus *Hydrachna* Muller attach to thorax and abdomen sternites and tergites under the elytra (Zawal 2002), while larvae of genus *Eylais* Latreille mostly hide under the beetle’s elytra (Zawal 2003b). In this study, we found three larvae of *Hydrachna* sp. attaching to the mesosternal area of the dytiscid host beetle *Eretesgriseus* (Fabricius, 1781) (Fig. 2).

**Figure 1.**
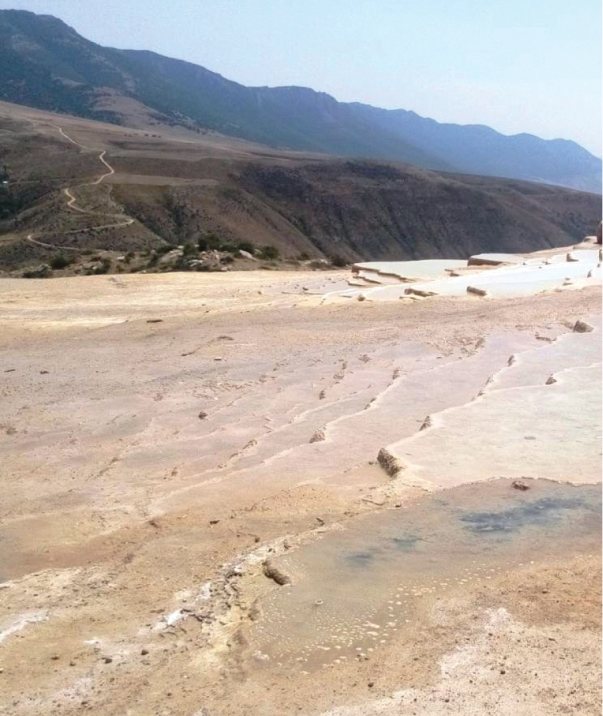
Photographs of sampling site.

**Figure 2.**
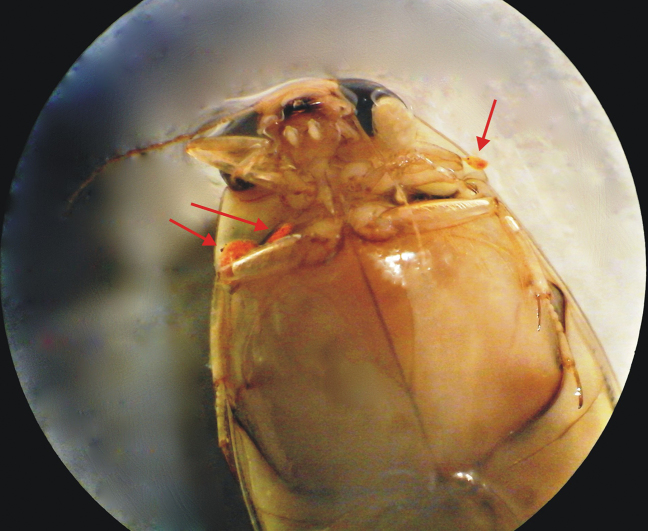
Larvae of *Hydrachna* sp. attached to *Eretesgriseus*.

## Material examined

The larvae were collected as parasites on *Eretesgriseus* from a volcanic area on a mountainside, nearly 1,840 meters above the sea level, Badab-e Soort) 36.3549N, 53.8565E (in Mazandarn province, northern Iran (Fig. 1). This natural site comprises two mineral hot springs, one with sour and the other with salty water. Over thousands of years, flowing water from these springs has formed numbers of red, orange, and yellow staircase pools each filled with some amount of mineral water. We collected the host beetle with a net from one of the lower pools as it was swimming.

All small larvae (0.15–0.40 mm) were detached from surfaces of the mesosternum of the beetle body (Fig. 2). They were cleared with lactic acid and mounted in Hoyer’s medium. Morphological observations, measurements, and illustrations were made using compound microscopes (Zeiss Axio Scope.A1) equipped with phase contrast optical systems and a camera lucida (Olympus BX51).

Idiosomal setae are named according to Prasad and Cook (1972):

**Cx-1-3** coxal plates,

**Hu** humeral seta,

**L** length,

**Lp1, Lp2** lateropropodosomal setae,

**Lh3** laterohysterosomal seta,

**Mh1, Mh2, Mh3, Mh4** mediohysterosomal setae,

**Mp1, Mp2** mediopropodosomal setae,

**n** number of specimens measured,

**P–1–5** pedipalp segments (trochanter, femur, genu, tibia and tarsus),

**I–Leg-1–5** first leg, segments 1–5 (trochanter, femur, genu, tibia and tarsus) i.e.,

**III– Leg–3** genu of third leg,

**W** width.

All measurements are given in micrometres (μm).

## Taxonomic account

### Superfamily HYDRACHNOIDEA

#### Family Hydrachnidae Leach, 1815

##### Genus *Hydrachna* Müller, 1776

###### Hydrachna

Taxon classificationAnimaliaTrombidiformesHydrachnidae

sp.

ac352e1a-6216-4cf8-8e87-20f7cb4908fb

Figs 3–10 , 11

####### Description.

**Figures 3–10.**
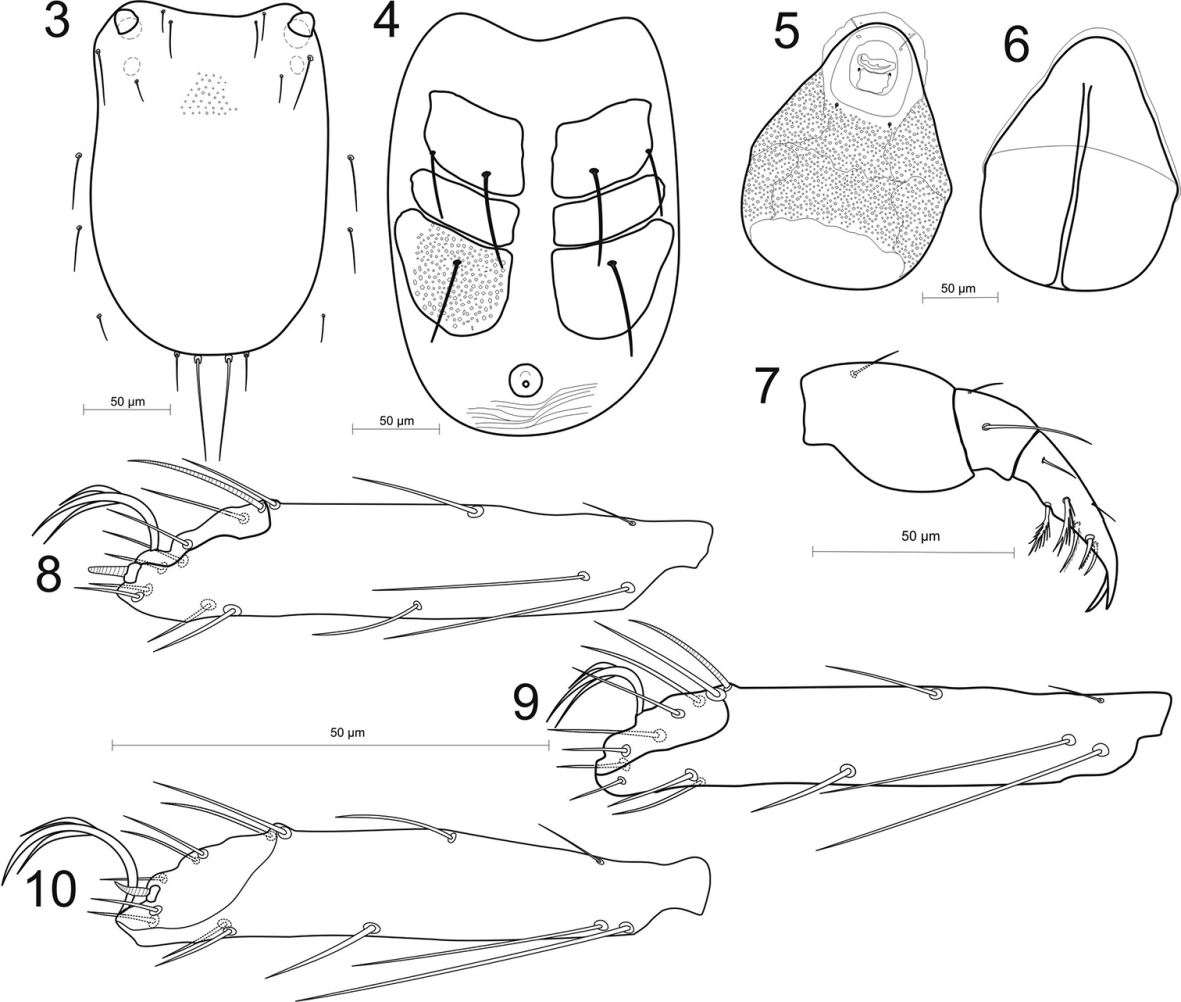
*Hydrachna* sp. **3** dorsal plate **4** ventral side **5** gnathosoma ventral side **6** gnathosoma dorsal side **7** pedipalp **8** I-leg-5 **9** II-leg-5 **10** III-leg-5.

**Figure 11.**
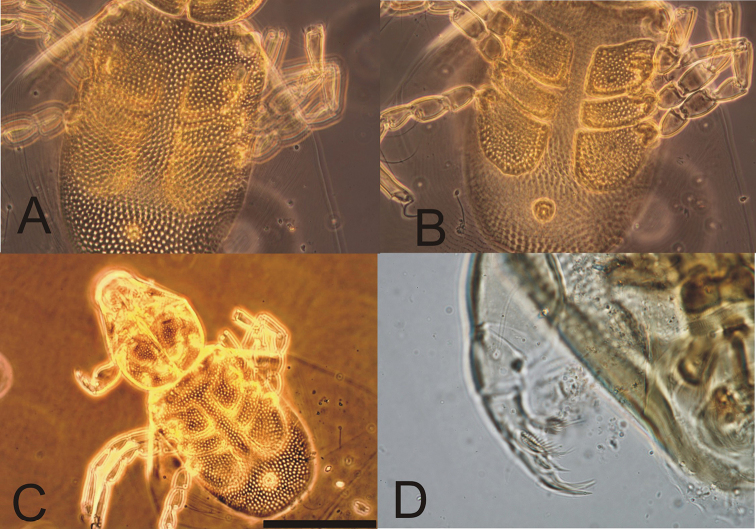
Photographs of *Hydrachna* sp. from *Eretesgriseus***A** dorsal plate **B** ventral side **C** dorsal view **D** pedipalp.

The idiosoma are oval, with the integument striated, and the dorsal plate is very large, covering the whole idiosoma of unengorged larva, the integument pointed and with a concave anterior edge (Figs 3, 11). There are four pairs of setae on the dorsal plate (Mp1, Lp1, Lp2, Hu). The basal bodies of Mp2 on dorsal plate invisible; setae Mh1, Mh2, Mh3 located on soft integument (Fig. 4). There are three pairs of coxal plates located on the proximal half of the idiosoma, and all are wider than long. Median edges of coxa I and III almost the same length and two time longer then coxa II. The anterior coxa bears two setae, the medial coxa is without seta, and the posterior coxa has one seta. The excretory pore plate is very large and is located behind of coxal plates (Figs 5, 11). Gnathosoma short, strongly tapering forward; gnathosomal sucker large, discoid with corrugated borders (Figs 5, 6). Pedipalps relatively short and thin: femur stocky with strongly convex ventral margin and one seta; genu with two setae and concave ventral margin; tibiotarsus relatively long with two claws the same size, weakly bent, five tibiotarsal spines, four of them pinnate (Fig. 7). Trochanters of all legs with one seta, all femora with four setae and with one swimming seta on I and II and two swimming setae on III femora. Genu I with five setae including two swimming setae, genu II and III with four setae including one swimming seta. All tibiae with five setae including one swimming seta, and with one solenidium. Tarsi each have 14 setae including two swimming setae, tarsi I and II have one solenidium, and tarsi I and III have one eupathidium (Figs 8–10).

####### Measurements.

In µm, n = 3. Dorsal plate: L/W 250–254/162–157; coxal plates: Cx-1 L 40–45, Cx-2 L 20–22, Cx-3 42–44; excretory pore plate L/W 17–18/16–17; gnathosoma; L/W 173–176/138–140; diameter of sucker ring 71–73; pedipalpal segments (P-1–3) L: 8–9, 36–38, 39–42; leg segments L: I-leg 1–5: 18–19, 37–39, 32–34, 38–40, 67–69; II-leg 1-5: 20–21, 32–34, 29–30, 37–39, 68–70; III-leg 1-5: 28–29, 29–30, 27–28, 38–40, 61–64.

####### Remarks.

The larva of *Hydrachna* sp. is most similar to larvae of *H.processifera* described by Wainstein (1980) as a *H.inermis* (Aykut et al. 2018). It is similar in the shape of coxal plates, the discoidal hypostomal sucker, the tibiotarsus relatively long with two claws the same size, weakly bent; five tibiotarsal spines the same size. It is different by the presence of a eupathidium on tarsus leg-2; localisation the Mh1, Mh2, and Mh3 setae outside of the dorsal plate on soft integument, and the presence of a very large excretory pore plate. The last two features are very strange and different from all other species of *Hydrachna*. These differences indicate the probability of a separate subgenus to which the described larva would belong.

Thor (1916) split the genus *Hydrachna* into five subgenera: *Hydrachna* s. str., *Anahydrachna*, *Diplohydrachna*, *Schizohydrachna*, and *Monochydrachna*; subsequently he synonymised *Monochydrachna* with *Hydrachna* s. str., and *Schizohydrachna* with *Diplohydrachna*, and established two more subgenera: *Rhabdohydrachna* and *Scutochydrachna* (Thor 1925). Davids et al. (2007) stated the differences between these subgenera were not clear and he abolished the division into subgenera.

At the current level of research, we propose to leave the taxonomy of the genus *Hydrachna* without sub-division, indicating the existence of greater morphological differentiation. Relationships within the genus of *Hydrachna* should be recognised on the basis of molecular studies and a decision on the possible splitting the genus into subgenera should be made. Up to now six species of *Hydrachna* were recorded from Iran (*H.cruenta*, *H.skorikowi*, *H.sepasgozariani*, *H.* cf. v*aillanti*, *H.sistanica*, *H.globosalacerata*), and two of them (*H.sepasgozariani*, *H.* cf. v*aillanti*) belong to the *Hydrachnaprocessifera* group of species (Pešić and Saboori 2007; Pešić et al. 2012, 2014). Larvae were described only for *H.cruenta*, *H.skorikowi*, and *H.globosa* (Wainstein 1980). The morphology of this larva and its parasitism on Dytiscidae show plausible grounds for it belonging to the *H.processifera* group of species and possibly to one of the two species from Iran (*H.sepasgozariani* or *H.* cf. v*aillanti*) for which the larvae are still not described. On the other hand, the differences in morphology (localisation the Mh1, Mh2, Mh3 setae outside of dorsal plate, on soft integument and very large excretory pore plate) indicate that it could belong to another species.

## Supplementary Material

XML Treatment for Hydrachna
